# The Generation of Human iPSC Lines from Three Individuals with Dravet Syndrome and Characterization of Neural Differentiation Markers in iPSC-Derived Ventral Forebrain Organoid Model

**DOI:** 10.3390/cells12020339

**Published:** 2023-01-16

**Authors:** Valery Zayat, Zuzanna Kuczynska, Michal Liput, Erkan Metin, Sylwia Rzonca-Niewczas, Marta Smyk, Tomasz Mazurczak, Alicja Goszczanska-Ciuchta, Pawel Leszczynski, Dorota Hoffman-Zacharska, Leonora Buzanska

**Affiliations:** 1 Department of Stem Cell Bioengineering, Mossakowski Medical Research Institute, Polish Academy of Sciences, 02-106 Warsaw, Poland; 2 Medical Genetics Department, Institute of Mother and Child, 01-211 Warsaw, Poland; 3 Faculty of Biology, Institute of Genetics and Biotechnology, University of Warsaw, 02-106 Warsaw, Poland

**Keywords:** *SCN1A*-related disorders, Dravet syndrome, Panayiotopoulos syndrome, Nav1.1 haploinsufficiency, stem cell reprogramming, organoids

## Abstract

Dravet syndrome (DRVT) is a rare form of neurodevelopmental disorder with a high risk of sudden unexpected death in epilepsy (SUDEP), caused mainly (>80% cases) by mutations in the *SCN1A* gene, coding the Nav1.1 protein (alfa-subunit of voltage-sensitive sodium channel). Mutations in *SCN1A* are linked to heterogenous epileptic phenotypes of various types, severity, and patient prognosis. Here we generated iPSC lines from fibroblasts obtained from three individuals affected with DRVT carrying distinct mutations in the *SCN1A* gene (nonsense mutation p.Ser1516*, missense mutation p.Arg1596His, and splicing mutation c.2589+2dupT). The iPSC lines, generated with the non-integrative approach, retained the distinct *SCN1A* gene mutation of the donor fibroblasts and were characterized by confirming the expression of the pluripotency markers, the three-germ layer differentiation potential, the absence of exogenous vector expression, and a normal karyotype. The generated iPSC lines were used to establish ventral forebrain organoids, the most affected type of neurons in the pathology of DRVT. The DRVT organoid model will provide an additional resource for deciphering the pathology behind Nav1.1 haploinsufficiency and drug screening to remediate the functional deficits associated with the disease.

## 1. Introduction

Dravet syndrome (DRVT, OMIM 607208) is a severe form of developmental and epileptic encephalopathy, which typically appears during the first year of life in an otherwise healthy infant, often triggered by fever [1]. After the first febrile seizure (tonic-clonic/clonic), seizures can happen without a fever. With the progression of the disease, different types of seizures and status epilepticus may also be experienced, which may lead to permanent brain damage or death (SUDEP among DRVT patients has the highest rate among epilepsy patients). The majority of cases of DRVT (more than 80%) are caused by mutations in the *SCN1A* gene (Sodium Voltage-Gated Channel Alpha Subunit 1; OMIM 182389), which mainly occur *de novo*. Until now more than one thousand mutations in this gene have been described (*SCN1A* mutation database; http://scn1a.caae.org.cn/index.php; accessed on 4 October 2022). The *SCN1A* gene encodes the alfa-subunit of the voltage-gated sodium channel called Nav1.1. Nav1.1 is expressed primarily in the central nervous system (CNS). It controls the influx of sodium ions into the cells and is mainly responsible for the generation and propagation of neuronal action potentials. Mutations in the *SCN1A* are linked to a broad spectrum of epileptic phenotypes with different severity [2]. An exact pathomechanism leading to *SCN1A*-related disorders has not been fully elucidated. However, the phenotypes are related to the functional effect of underlying loss or gain of function mutation (LOF vs. GOF). DRVT, the most common *SCN1A*-related disorder as well as the milder form of genetic epilepsy with febrile seizures plus (GEFS+, OMIM 604403), is caused by LOF mutations, but the most severe non-Dravet developmental and epileptic encephalopathies (DEE6B, OMIM 619317) are triggered by GOF mutations [2]. Some of the latest research points to the theory that one of the ways the mutation could act is by impairing the activity of the nervous system through a type of inhibitory type neurons (GABA neurons) leading to no inhibitory signals being transmitted. This would result in the overactivation of the nervous system, the transmission of signals uncontrollably, and the development of seizures. There may be other ways in which the *SCN1A* gene mutations alter the functioning of the sodium channels and the nervous system, leading to the development of seizures, but the molecular mechanisms underlying these events are not known.

One of the major obstacles in deciphering the mechanism of epileptic encephalopathies, including DRVT, is the lack of an in vitro model that truly recapitulates the complexity of these complex diseases [3]. The emerging field of human induced pluripotent stem cells (hiPSCs) has enabled the differentiation of iPSCs into different cell types. The hiPSCs from patients and healthy individuals together with the development of the CRISPR/Cas9 gene modification technology have vastly improved the modeling of human diseases [4]. Several reports showed that DRVT has been modeled using the neuronal model derived from pluripotent stem cells (PSCs) [5]. Schuster et al. used iPSC-derived neural progenitor cells (DRVT-iPSC NPC) and iPSC GABAergic cells (DRVT-iPSC GABA) from healthy controls and three DRVT patients with different *SCN1A* mutations. Transcriptomic analysis revealed the misregulation of molecular pathways specific to brain regions that are relevant for clinical features in DRVT. Moreover, electrophysiological abnormalities were recorded in the DRVT-iPSC GABA lines carrying *SCN1A* variants consistent with a decreased sodium current density and impaired excitability of inhibitory interneurons [6]. Higurashi et al. performed a patch-clamp analysis of iPSC-derived neurons from a DRVT patient with a p.Arg1645 * mutation in the *SCN1A* gene [7]. The results revealed impairments in action potential generation in response to sustained current injection, confirming previous studies from in vitro rodent epilepsy models with *SCN1A* mutation [8,9]. To date, the brain organoid system for DRVT has not been established.

Here, the iPSC lines were generated from the dermal fibroblast samples obtained from three individuals with DRVT and Panayiotopoulos syndrome (PS) carrying distinct mutations in the *SCN1A* gene. The resulting iPSC lines were used to generate ventral forebrain organoids with the subsequent characterization of their transcriptional profile. The Gibco™ Episomal hiPSC line was used as a positive control due to its well-established pluripotency and differentiation potential. The connection between mitochondrial biogenesis and neural differentiation during early brain development in 3D culture conditions was also investigated.

## 2. Materials and Methods

### 2.1. Cell Culture and Reprogramming

Human dermal fibroblast (HDF) lines were established from skin punch biopsies obtained from three patients [10]. Fibroblasts from DRVT/PS patients (DS 1-3) were reprogrammed using the Cytotune^®®^ iPS 2.0 kit (Thermo Fisher Scientific, Waltham, MA, USA) following the manufacturer’s protocol with minor modifications [11].

The HDF lines were cultured in DMEM (Sigma-Aldrich, Burlington, MA, USA), 10% fetal bovine serum (Thermo Fisher Scientific, Waltham, MA, USA), 2 mM GlutaMAX™ (Thermo Fisher Scientific, Waltham, MA, USA), 1% penicillin/streptomycin (Thermo Fisher Scientific, Waltham, MA USA) in a humidified atmosphere with 5% CO_2_ at 37 °C and passaged using TrypLE™ Express (Thermo Fisher Scientific, Waltham, MA, USA) at a ratio of 1:5 when 80–90% confluent.

All three iPSC lines generated from somatic samples of DRVT/PS patients were maintained on Vitronectin-XF™ (STEMCELL Technologies, Vancouver, BC, Canada) coated plates in Essential E8™ Medium (Thermo Fisher Scientific, Waltham, MA USA) and passaged as clumps every 3–4 days using 0.5 mM EDTA solution (Sigma-Aldrich, Burlington, MA, USA) at a ratio of 1:3 when 60–80% confluent.

### 2.2. Sanger Sequencing

The presence of heterozygous *SCN1A* variants was confirmed in dermal fibroblasts and iPSCs lines derived from DRVT/PS patients by bidirectional sequencing of PCR products using primers flanking each *SCN1A* variant. The standard conditions for the BigDye Terminator v3.1 Cycle Sequencing Kit (Thermo Fisher Scientific, Waltham, MA, USA) were used.

### 2.3. RNA Isolation, Quantitative Real-Time PCR (RT-PCR)

Total RNA from the donor dermal fibroblasts, DRVT/PS patients’ iPSC lines, patient and healthy iPSC-derived (Gibco^TM^ Episomal hiPSC Line, Thermo Fisher Scientific, Waltham, MA USA) ventral forebrain organoid samples were isolated using a miRNeasy micro kit (Qiagen, Hilden, Germany) following the manufacturer’s protocol. Genomic DNA was eliminated from RNA samples by a DNAse treatment using a Clean-Up RNA Concentrator kit (A&A Biotechnology, Gdansk, Poland). For RT-PCR, 1 μg RNA was reverse-transcribed into cDNA in the RT reaction using High Capacity cDNA Synthesis Kit (Thermo Fisher Scientific, Waltham, MA USA). cDNA was used to analyze relative gene expression (RT-PCR) prepared with PowerUp™ SYBR^®®^ Green Master Mix (Thermo Fisher Scientific, Waltham, MA USA) onto a 96-well plate for LightCycler^®®^ 96 (Roche Diagnostics GmbH, Basel, Switzerland). The expression of genes was analyzed at the mRNA level by the RT-PCR method in the presence of specific primers for genes (Table 1). The panel of genes includes stem cell pluripotency: *NANOG* (Nanog Homeobox), *POU5F1* (Oct4, Octamer-Binding Protein 4), *SOX2* (SRY, Sex-Determining Region Y, Box 2); GABAergic neuronal markers: *GAD67* (Glutamic Acid Decarboxylase 67) and *ABAT* (4-Aminobutyrate Aminotransferase); ventral forebrain interneuronal markers: *DLX2* (Distal-less Homeobox 2), *LHX6* (LIM Homeobox 6), and *SST* (Somatostatin); mitochondrial biogenesis markers: *NRF1* (Nuclear Respiratory Factor 1), *PPARGC1A (*Peroxisome Proliferator-activated Receptor Gamma Coactivator 1-alpha, PGC-1α), and *TFAM* (Mitochondrial Transcription Factor A). To determine the relative expression of the pluripotency markers relative to *GAPDH* (Glyceraldehyde-3-phosphate Dehydrogenase), the 2^−∆Ct^ method was used. For the relative gene expression of the ventral forebrain organoids, the 2^−∆∆Ct^ method was used. *GAPDH* was used to normalize the gene expression data.

### 2.4. Immunofluorescence

Cells were fixed in 4% formaldehyde for 12 min at room temperature (RT) and pre-incubated in 0.3% TritonX100 diluted in PBS at RT. After washing in PBS, the cells were permeabilized and blocked in 10% NGS with 0.2% Triton 100 for 1 h at RT. Primary antibodies mouse anti-Oct4 (Octamer-Binding Protein 4, Santa Cruz Biotechnology, Dallas, TX, USA), mouse anti-SOX2 (SRY, Sex-Determining Region Y, Box 2, Invitrogen, Waltham, MA USA), rabbit anti-NANOG (Nanog Homeobox, Cell Signaling Technology, Danvers, MA, USA), mouse anti-SSEA4 (Stage-specific Embryonic Antigen 4, Millipore, Burlington, MA, USA), rabbit anti-Ki67 (Marker Of Proliferation Ki-67, Millipore, Burlington, MA, USA), mouse anti-CD73 (Ecto-5′-nucleotidase, Santa Cruz, Dallas, TX, USA), and mouse anti-SeV (Sendai virus, Invitrogen, Waltham, MA USA) were diluted in pre-incubation buffer and incubated at 4 °C overnight. The secondary antibodies goat anti-Mouse IgG2a (Alexa Fluor^®®^ 488), goat anti-Mouse IgG2b (Alexa Fluor^®®^ 488), goat anti-Mouse IgG3 (Alexa Fluor^®®^ 488), goat anti-Rabbit IgG H+L (Alexa Fluor^®®^ 546), goat anti-Mouse IgG1 (Alexa Fluor^®®^ 546) (1:500; Thermo Fisher Scientific, Waltham, MA USA) were applied alone or in appropriate combinations for 1.5 h at room temperature in the dark (Table 2). The nuclear marker, Hoechst diluted 1:100 was incubated for 10 min at room temperature and specimens were mounted onto microscope slides using Dako mounting medium. Visualizations were performed on Zeiss LSM 780 Elyra PS.1 in the Laboratory of Advanced Microscopy Techniques (MMRI PAS, Warsaw, Poland).

### 2.5. Differentiation into Three Germ Lineages

To assess the pluripotency of the iPSC lines generated from the samples of DRVT/PS donors, directed differentiation of iPSC lines to all three germ layers was performed using STEMdiff™ Trilineage Differentiation Kit (STEMCELL Technologies, Vancouver, BC, Canada) according to the manufacturer’s protocol. The following fluorochrome-conjugated antibodies specific for early differentiation for each germ layer were used: Ectoderm: SOX1 (SRY-Box Transcription Factor 1) and OTX2 (Orthodenticle Homeobox 2); Mesoderm: Brachyury (T-box Transcription Factor T) and HAND1 (Heart And Neural Crest Derivatives Expressed 1); Endoderm: SOX17 (SRY-Box Transcription Factor 17) and GATA4 (GATA Binding Protein 4) (Table 2). All antibodies were a part of the STEMdiff™ Trilineage Differentiation Kit (STEMCELL Technologies, Vancouver, BC, Canada).

### 2.6. Generation of Ventral Forebrain Organoids

The generation of ventral forebrain organoid culture was performed according to well-established protocols [13,15] with minor modifications. The iPSC colonies were dissociated from the plates using first 0.5 mM EDTA solution (Sigma-Aldrich, Burlington, MA, USA) and then Accutase^®®^ solution (Sigma-Aldrich, Burlington, MA, USA). The dissociated cells were seeded into AggreWell™800 plate (STEMCELL Technologies, Vancouver, BC, Canada) at the concentration of 10,000 cells/microwell in hPSC medium containing Essential 8™ Medium (Thermo Fisher Scientific, Waltham, MA USA) supplemented with the ROCK inhibitor Y-27632 (10 μM; Selleckchem, Houston, TX, USA). After 48 h, the aggregates of cells were transferred into the low-attachment 60 mm plates coated with Poly (2-hydroxyethyl methacrylate) (Sigma-Aldrich, Burlington, MA, USA). Starting on day 6, the hPSC medium was changed to a neural induction medium containing Essential 6™ Medium (Thermo Fisher Scientific, Waltham, MA USA) supplemented with the two SMAD inhibitors, dorsomorphin (DM; 5 μM; Sigma-Aldrich, Burlington, MA, USA) and SB-431542 (SB; 10 μM, Tocris, Bristol, UK). The medium was supplemented with the Wnt pathway inhibitor IWP-2 (5 μM; Selleckchem, Houston, TX, USA) from day 10. On day 14, the cell aggregates were transferred into the low-attachment 96-well plates coated with Poly(2-hydroxyethyl methacrylate) (Sigma-Aldrich, Burlington, MA, USA) in a neural differentiation medium containing Neurobasal™-A Medium (Thermo Fisher Scientific, Waltham, MA USA), B-27 supplement without vitamin A (Thermo Fisher Scientific, Waltham, MA USA), GlutaMax (Thermo Fisher Scientific, Waltham, MA USA, 1:100), penicillin, and streptomycin (Thermo Fisher Scientific, Waltham, MA USA, 1:100) and supplemented with the growth factors EGF (20 ng/mL; R&D Systems, Minneapolis, MN, USA) and FGF2 (20 ng/mL; R&D Systems, Minneapolis, MN, USA) until day 18. From days 19 to 30, the differentiation medium was supplemented with the Hedgehog pathway activator SAG (100 nM; R&D Systems, Minneapolis, MN, USA). Starting on day 25, the medium was replaced every other day. On day 33, the differentiation medium was supplemented with the growth factors BDNF (20 ng/mL; Peprotech, Rocky Hill, NJ, USA) and NT3 (20 ng/mL; Peprotech, Rocky Hill, NJ, USA) with medium changes every other day until day 60.

### 2.7. Karyotyping

Chromosome preparation of iPSC lines was performed as described previously [16]. Twenty metaphases were analyzed for each cell line. To exclude clinically relevant copy number variants array, comparative genomic hybridization (aCGH) was performed using the 60K CytoSure Constitutional v3 microarray (Oxford Gene Technology, Oxford, UK) according to the manufacturer’s instructions.

### 2.8. Mycoplasma

The presence of mycoplasma in iPSC lines was analyzed on cell culture supernatants using MycoAlert™ Mycoplasma Detection kit (Lonza, Basel, Switzerland) according to the manufacturer’s protocol.

### 2.9. Ethical Approval

All procedures were performed according to the Declaration of Helsinki and written informed consent was obtained from all patients or their legal representatives. The skin punch biopsies were approved by the ethical committee of the Institute of Mother and Child in Warsaw, Poland number 22/2020 from 21 May 2020.

### 2.10. Statistical Analysis

GraphPad Prism 9.0. was used to perform statistical analysis. Kolmogorov-Smirnov was used as a normality test. After checking if the distribution was normal, test samples were compared with One-way ANOVA followed by Tukey’s Multiple Comparison Test: (*) *p* < 0.05, (**) *p* < 0.005, (***) *p* < 0.0005. Results represent three independent experiments, each in at least three replicates. Results presented in the graphs were shown as mean with standard deviation (SD).

## 3. Results

### 3.1. Isolation of Human Dermal Fibroblasts and Generation of iPSC Lines

Skin fibroblasts were isolated from the patients carrying three different *SCN1A* mutations. c.2589+2dupT splicing and p.Ser1516* are nonsense mutations, both *de novo* identified in patients diagnosed with DRVT. p.Arg1596His is a missense mutation found in a familial case of febrile seizures (FS/FS+) and Panayiotopoulos syndrome (Table 3).

The c.2589+2dupT splicing mutation is most likely pathogenic. It gives the same sequence change as c.2589+3A>T, in all probability causing the Ex14 deletion (p.Val806_Leu863del) [17] (https://scn-portal.broadinstitute.org/; accessed on 4 October 2022).

**Figure 1:**
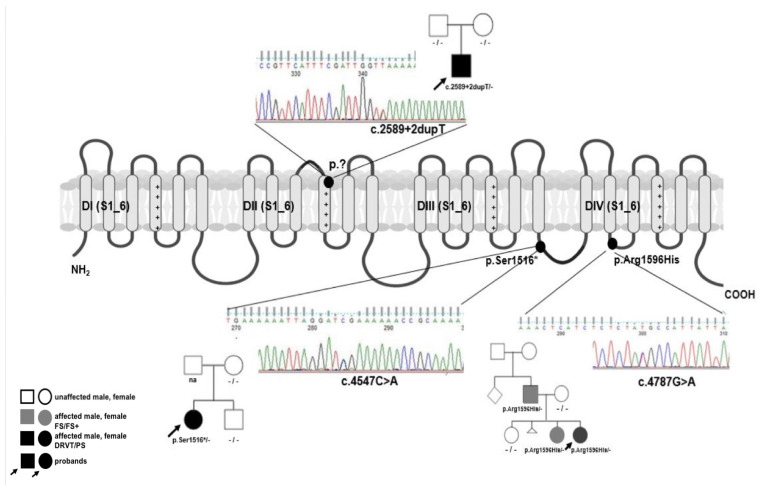
Schematic localization of *SCN1A*/Nav1.1 variants under analysis. Two truncating mutations nonsense c.4547C>A/p.Ser156* and splicing c.2589+2dupT/p.? arose *de novo* and both probands were diagnosed with DRVT. Missense c.4787G>A/p.Arg1596His is hereditary giving a heterogenous clinical picture in the family from febrile seizures/febrile seizures plus to severe syndrome primarily diagnosed as DRVT but finally as Panayiotopulos syndrome [18]. Sanger sequencing chromatograms are presented for all variants. Created with BioRender.com.

Dermal fibroblasts, isolated from all three patients, were infected with the viral cocktail containing *KLF4*, *OCT4*, *SOX2*, and *c-MYC* reprogramming genes. Following the reprogramming, the presence of original mutations was confirmed in diagnostic PCR analysis in all iPSC lines generated from the three donors. All iPSC lines were heterozygous for the respective mutations (Figure 1).

### 3.2. Classification and Validation of iPSC Lines Generated from DRVT/PS Donors

**Figure 2:**
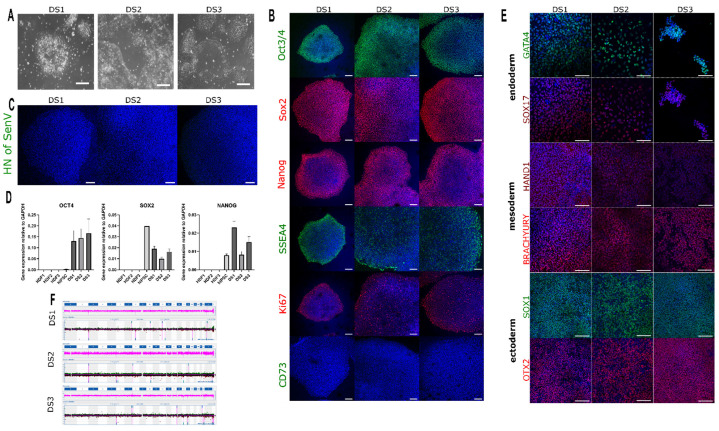
Characterization and validation of the iPSC lines derived from samples of donors diagnosed with DRVT/PS. (**A**) Bright-field image of PSC lines generated from samples of three donors with DRVT/PS; (**B**) Immunocytochemistry of three iPSC lines expressing the pluripotency markers OCT4, SOX2, NANOG, and SSEA4; (**C**) Sendai virus was not detected in all iPSC lines; (**D**) RT-PCR of pluripotency markers *OCT4*, *SOX2*, and *NANOG* in iPSC lines. Results shown in brackets were obtained as mean (±SD); (**E**) Differentiation into three germ lineages showed normal expression of lineage-specific markers: Ectoderm: SOX1 and OTX2; Mesoderm: Brachyury (T) and HAND1; Endoderm: SOX17 and GATA4; (**F**) Karyotyping of all iPSC lines showed normal genotype. Results represent three independent experiments. The 2^−∆Ct^ method was used to determine the gene expression of the pluripotency markers for RT-PCR. Scale bars: 100 μm. HDF: human dermal fibroblasts.

Based on representative iPSC morphology and growth rate, only one iPSC line from each donor was chosen for further validation analysis (Figure 2A). Endogenous expression of the pluripotency markers OCT4, SOX2, NANOG, and SSEA4 was demonstrated in all three patients’ iPSC lines by immunocytochemistry, whereas the mesenchymal stem cell marker CD73 showed no expression (Figure 2B). Sendai virus used for conversion was not detected in the three iPSC lines by immunocytochemistry (Figure 2C). All iPSC lines expressed pluripotency markers *OCT4*, *SOX2*, and *NANOG*, whereas HDFs of origin showed no expression (Figure 2D). The differentiation potential of the converted iPSC lines into the three germ layer markers was analyzed.

The analysis revealed the expression of the ectodermal markers SOX1 and OTX2, the mesodermal markers Brachyury (T) and HAND1 as well as the endodermal markers SOX17 and GATA4 by immunocytochemistry in all three converted iPSC lines (Figure 2E). The genomic integrity was examined through karyotyping and revealed no acquired genomic abnormalities in the three iPSC lines (Figure 2F). In addition, all analyzed iPSC lines were free of Mycoplasma contamination (Table 4).

In summary, all iPSC lines generated from donors with DRVT/PS containing *SCN1A* mutations were validated for pluripotency markers, differentiation potential, and the absence of chromosomal aberrations and used to investigate the relationship between mitochondrial biogenesis and neurogenesis in the ventral forebrain organoid model.

### 3.3. Generation of Ventral Forebrain Organoids

**Figure 3:**
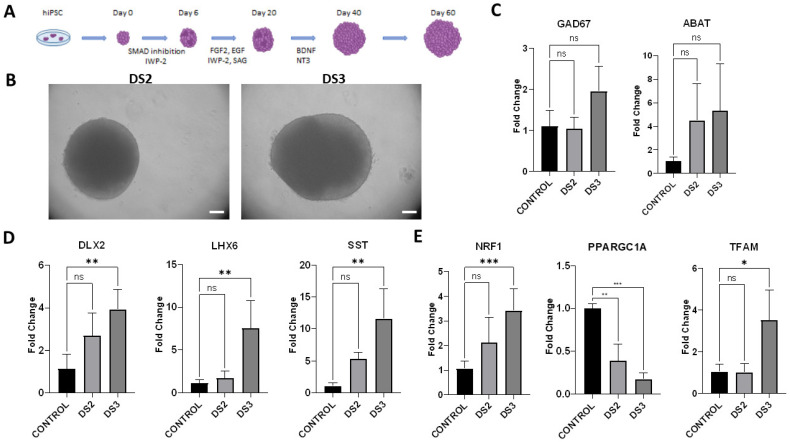
Transcriptional profile of ventral forebrain organoids generated from iPSC lines derived from patients with distinct SCN1A mutations by RT-PCR. (**A**) Generation of ventral forebrain organoids scheme; (**B**) Bright-field image of ventral forebrain organoids generated from DS2 and DS3 iPSC lines (d60); (**C**) Gene expression levels of GABAergic neuronal markers GAD67 and ABAT; (**D**) The expression levels of ventral forebrain interneuronal markers DLX2, LHX6, and SST; (**E**) Gene expression of mitochondrial biogenesis markers NRF1, PPARGC1A and TFAM. GAPDH was used to normalize the gene expression data. Control: ventral forebrain organoids generated from iPSC line derived from a healthy donor. Results represent three independent experiments, at least 5 organoids were used for each replicate. Brackets show statistical significance between all samples (one-way ANOVA): (*), *p* < 0.05; (**), *p* < 0.005; (***), *p* < 0.0005. Results shown in brackets were obtained as mean (±SD). Scale bars, 300 μm.

Since DRVT/PS pathophysiology implicates GABAergic neuron defects as the primary cause of the disease mechanism, the ventral forebrain organoid culture was generated. The iPSC lines carrying distinct *SCN1A* mutations were used to generate ventral forebrain organoids with the subsequent characterization of their transcriptional profile (Figure 3A–E).

Two iPSC lines were used, one established from a donor with a familial case of febrile seizures (FS/FS+)/Panayiotopoulos syndrome carrying a missense p.Arg1596His (DS2) mutation and another from a donor with DRVT carrying a p.Ser1516* (DS3 line) nonsense mutation. These lines reflected the severity of the symptoms in the respective donors, from mild to more severe clinical picture. The expression of the GABAergic neuronal markers in 60-day ventral forebrain organoid culture generated from DS2 and DS3 iPSC lines was compared to healthy control. The expression of GAD67 and ABAT was mildly elevated in both organoid cultures, yet this was not statistically significant compared to healthy control. Interestingly, the GAD67 levels in DS3 organoids were mildly increased compared to the DS2 culture, perhaps reflecting the degree of severity of the underlying pathology. The ventral forebrain markers *DLX2*, *LHX6*, and *SST* were increased in DS3 organoids compared to healthy control, whereas the changes in expression levels in DS2 organoids were not statistically significant. A significant decrease in the expression levels of the main regulator of mitochondrial biogenesis *PPARGC1A* was shown both in DS2 and DS3 organoid cultures compared to healthy control. The markers of mitochondrial biogenesis *NRF1* and *TFAM* were significantly upregulated in DS3 organoids compared to healthy control. The expression levels of *TFAM* in DS2 organoids were not statistically significant compared to healthy control.

In summary, the results showed a significant increase in the expression levels of ventral forebrain interneuronal markers and mitochondrial biogenesis markers in DS3 ventral forebrain organoids compared to healthy control, which could be related to more severe clinical manifestations of the symptoms.

## 4. Discussion

The first evidence that the pathology underlying DRVT occurs due to the reduced excitability of GABAergic interneurons, leading to neuronal network hyperexcitability was discovered in animal models [8]. Subsequently, several research groups demonstrated the involvement of GABAergic neurons in functional deficits associated with DRVT [6,7,19,20]. It was also shown that the missense mutations identified in GEFS+ and DRVT patients exhibit a common pathogenic mechanism for both disorders [21]. The mutations in the *SCN1A* gene are linked to a broad spectrum of epileptic phenotypes, which differ in the clinical presentation of symptoms. In this work, the ventral forebrain organoids were generated from iPSC lines established from the samples of donors afflicted with DRVT/PS carrying distinct *SCN1A* mutations. The DS1 and DS3 lines were generated from the samples of patients diagnosed with more severe forms of developmental and epileptic encephalopathies, the DRVT, whereas the DS2 line was generated from the milder form of genetic epilepsy with febrile seizures. The intent of the study was to compare transcriptional profiles of the generated ventral forebrain organoids established from samples of patients with opposite positions on the severity scale of the symptoms. Since DS1 and DS3 lines exhibited a more severe clinical phenotype, only the DS3 line was chosen for subsequent analysis. Birey et al. showed the expression of GAD67 and SST at day 109 in the ventral forebrain organoid culture established from iPSC lines derived from healthy donors and patients with Timothy syndrome [13]. Here, the expression of GABAergic neuronal markers was compared in 60-day ventral forebrain organoids generated from two iPSC lines, one established from a donor with a familial case of febrile seizures (FS/FS+)/Panayiotopoulos syndrome carrying a missense p.Arg1596His (DS2) mutation and another from a donor with DRVT carrying a p.Ser1516* (DS3) nonsense mutation. Grown under feeder-free conditions, the Gibco™ Episomal hiPSC line was used as a positive control for all reprogramming procedures and downstream experiments. It is a zero-footprint, viral-integration-free cell line, generated using a cocktail of reprogramming factors. While the source of the somatic cells used for reprogramming has an impact on the residual epigenetic signature, the intent of the study was to use a well-established bona fide iPSC control line of the highest quality as a gold standard for all newly derived hiPSC lines.

Although the expression of some genes was mildly elevated in DS2 organoids, the differences in comparison to the healthy control were not statistically significant. Similarly, the expression of genes involved in the synthase of GABA such as *GAD67,* and catabolism of GABA such as *ABAT* was also not significantly increased in DS3 organoids. Interestingly, in the DS3-derived ventral forebrain organoids, the expression of *SST* was significantly increased compared to the control. In contrast, the alterations of excitability in SST-expressing interneurons were reported in the neocortex in a mice model of DRVT [22].

The significant upregulation of *DLX2* involved in the development of GABAergic neurons was observed in DS3 organoids in comparison to the healthy control. As the material in this work was collected from 60-day organoids, it is plausible to speculate that *SCN1A* mutations affect the inhibitory neurons after cell maturation since the sodium channel Nav1.1 is involved in signal transmission. The developed interneurons present in the older ventral forebrain organoids might display the altered phenotype associated with DRVT. In future work, the analysis of electrophysiology should be investigated to address the DRVT-linked alterations.

In the mice model of DRVT, the GABAergic inhibitory neurons had reduced viability as the result of the heterozygous deletion of Nav1.1 channels in the forebrain, but the impact on the sodium current in the hippocampus was more severe compared to the cerebral cortex [23]. This suggests that in the cerebral cortex this type of affected GABAergic interneurons is functionally impaired or there is some compensatory system to account for that dysfunction [23]. This might explain why the reduced expression of GABAergic neuronal markers was not detected in the forebrain organoids derived from DRVT patients. In future research, the extensive characterization of the GABAergic inhibitory neurons in the ventral forebrain organoid model of DRVT at the mRNA and protein levels is required.

The expression of *LHX6*, which is involved in the migration of interneurons, was also increased in DS3 organoids in comparison to the healthy control. This thread requires further investigation of the interneurons’ migration in the assembloids of ventral and dorsal brain organoids.

Clinical presentation of *SCN1A* mutations ranges from febrile seizures and benign febrile seizures plus (FS/FS+) to severe epilepsy syndrome such as DRVT [17]. The complexity exhibited between the clinical presentation of DRVT and the location of *SCN1A* mutations suggests the involvement of multiple molecular pathways besides other developmental or environmental factors [24]. Early reports indicate that the scn1Lab mutant zebrafish model exhibits glucose and mitochondrial hypometabolism as contributing factors in the pathophysiology of DRVT [25]. At the same time, Graig et al. found two children that had pathological mutations in the *SCN1A* gene and defects in mitochondrial electron transport chain complex activity [26]. Since such studies indicate mitochondrial dysfunction in DRVT, the role of mitochondrial biogenesis was investigated in vitro models of early human neurogenesis. Peroxisome proliferator-activated receptor gamma coactivator 1-alpha (PGC1α) is considered to be the main regulator of the mitochondrial biogenesis process by interacting with nuclear respiratory factor 1 and 2 (NRF1, NRF2), resulting in the activation of mitochondrial transcription factor A (TFAM). A significant reduction in the expression levels of *PPARGC1A*, coding for PGC1α protein, was observed in DRVT/PS ventral forebrain organoids generated from both patients compared to healthy control. At the same time, there was a significant increase in the expression of *NRF1* and *TFAM* in the ventral forebrain organoid culture with DRVT causing mutation at day 60 compared to the healthy control. The organoid culture with the mutation causing FS/FS+/PS showed no significant increase in the *NRF1* and *TFAM* levels compared to the healthy control. In addition, there is evidence of a high concentration of PGC-1α in inhibitory neurons with high metabolic demand [27,28].

To date, DRVT was investigated in animal models or iPSC-derived neurons *in vitro*. Establishing brain-region organoids from donors afflicted with DRVT/PS allows for studying the effect of the disease in a more complex environment, which should display changes reflecting the degree of severity of the underlying pathology with DRVT exhibiting more severe functional deficits.

## 5. Conclusions

Developmental epilepsy and encephalopathy are considered to be among the most challenging disorders affecting early neural development. They are mainly drug-resistant and very difficult to treat (drug-resistant epilepsies). Thus, in attempts to treat such patients, there is a challenge in determining the optimal balance between the health benefits and tolerability of antiseizure medication. This problem may be solved by an understanding of the precise molecular mechanisms behind such neurodevelopmental disorders. In this work, the generated ventral forebrains were established from iPSC lines of donors diagnosed with DRVT/PS, and functional deficits in mitochondrial biogenesis were examined. This organoid model can be used to determine the stage of neural development at which the *SCN1A* gene begins expression, which would give the opportunities for intervention. Stem cell models of neurodevelopmental diseases provide an invaluable tool to investigate the molecular and functional pathology underlying these disorders.

## Figures and Tables

**Table 1:** The list of RT-PCR primers used for validation of *SCN1A* mutations, the pluripotency of reprogrammed iPSC lines, and neuronal gene expression in ventral forebrain organoids.

RT-PCR and Mutation Validation Primers	Target	Forward/Reverse Sequences (5′-3′)
Pluripotency markers	*OCT4* [12]	F: CCTGAAGCAGAAGAGGATCACC
		R: AAAGCGGCAGATGGTCGTTTGG
Pluripotency markers	*NANOG* [12]	F: GAACCTCAGCTACAAACAGG
		R: CGTCACACCATTGCTATTCT
Pluripotency markers	*SOX2* [12]	F: GTGGAAACTTTTGTCGGAGA
		R: TTATAATCCGGGTGCTCCTT
GABAergic neuronal markers	*GAD67* ^1^	F: TAGCGAGAACGAGGAAGCAG
		R: GCAGAAACAGGCTCGGCT
GABAergic neuronal markers	*ABAT* [13]	F: TGAAATACCCTCTGGAAGAG
		R: CAATCAGATCCTCCACCTC
Ventral forebrain interneuronal markers	*DLX2* [13]	F: ACGTCCCTTACTCCGCCAAG
		R: AGTAGATGGTGCGGGGTTTCC
Ventral forebrain interneuronal markers	*LHX6* [13]	F: CCGTCTGCAGGCAAGAACAT
		R: GACACACGGAGCACTCGAG
Ventral forebrain interneuronal markers	*SST* ^1^	F: AGTTTGACCAGCCACTCTCC
		R: GTACTTGGCCAGTTCCTGCTT
Mitochondrial biogenesis markers	*NRF1* ^1^	F: CACTTATCCAGGTTGGTACG
		R: CAGCCACGGCAGAATAAT
Mitochondrial biogenesis markers	*PPARGC1A* ^1^	F: CCCAGAGTCACCAAATGAC
		R: TCCAGAGAGTTCCACACTTA
Mitochondrial biogenesis markers	*TFAM* ^1^	F: CTCAGAACCCAGATGCAAA
		R: GCCACTCCGCCCTATAA
Housekeeping genes	*GAPDH* [14]	F: GAACGGGAAGCTTGTCATCAA
		R: ATCGCCCCACTTGATTTTGG
Mutation analysis/sequencing	*SCN1A*	F: TACTTCGCGTTTCCACAAGG
	*c.2589+2dupT*	R: GCTATGCAAGAACCCTGATTG
	*SCN1A*	F: CAAAAATCAGGGCCAATGAC
	*p.Arg1596His*	R: TGATTGCTGGGATGATCTTG
	*SCN1A*	F: GAGATTTGGGGGTGTTTGTC
	p.Ser1516*	R: GGATTGTAATGGGGTGCTTC

^1^ The primer pairs were designed using Primer-BLAST and the efficiency was tested using PowerUp SYBR Green Master Mix (Thermo Fisher Scientific, Waltham, MA USA). The primer efficiency was between 97 to 111% for all designed primers.

**Table 2:** The list of antibodies used for validation of pluripotency of reprogrammed iPSC lines and their differentiation potential.

Immunocytochemical Markers	Antibody	Dilution	Source
Pluripotency markers	Mouse anti-	0.1111111	Santa Cruz Biotechnology, Dallas, TX, USA, Cat# sc-5279, RRID: AB_628051
	OCT3/4		Cell Signaling Technology, Danvers, MA, USA, Cat# D73G4), RRID: AB_10559205
Pluripotency markers	Rabbit anti-	0.1111111	Thermo Fisher Scientific, Waltham, MA USA, Cat# MA1-014, RRID: AB_2536667
	NANOG		Millipore, Burlington, MA, USA, Cat# MAB4304, RRID: AB_177629
Pluripotency markers	Mouse anti-	0.1111111	R&D Systems, Minneapolis, MN, USA, Cat# NL557, RRID: AB 2157172
	SOX2		R&D Systems, Minneapolis, MN, USA, Cat# NL493, RRID: AB_2239879
Pluripotency markers	Mouse anti-	0.1805556	R&D Systems, Minneapolis, MN, USA, Cat# NL557, RRID: AB_2303012
	SSEA4		R&D Systems, Minneapolis, MN, USA, Cat# NL637, RRID: AB_2115853
Differentiation markers	Goat anti-	01:10	R&D Systems, Minneapolis, MN, USA, Cat# NL493, RRID: AB_1964595
	Otx2		R&D Systems, Minneapolis, MN, USA, Cat# NL637, RRID: AB_2195645
Differentiation markers	Goat anti-	01:10	Millipore, Burlington, MA, USA, Cat# AB9260, RRID: AB_2142366
	SOX1		Santa Cruz, Biotechnology, Dallas, TX, USA, Cat# sc-32299, RRID: AB_2033967
Differentiation markers	Goat anti-	01:10	Thermo Fisher Scientific, Waltham, MA USA, Cat# 14-6494-82, RRID: AB_2848274
	Brachyury		Thermo Fisher Scientific, Waltham, MA USA, Cat# A-21131, RRID: AB_2535771
Differentiation markers	Goat anti-	01:10	Thermo Fisher Scientific, Waltham, MA USA, Cat# A-21141, RRID: AB_2535778
	HAND1		Thermo Fisher Scientific, Waltham, MA USA, Cat# A-21151, RRID: AB_2535784
Differentiation markers	Goat anti-	01:10	Thermo Fisher Scientific, Waltham, MA USA, Cat# A-11035, RRID: AB_2534093
	GATA-4		Thermo Fisher Scientific, Waltham, MA USA, Cat# A-21123, RRID: AB_2535765
Differentiation markers	Goat anti-	01:10	
	SOX17		
Proliferation marker	Rabbit anti-	0.7361111	
	Ki-67		
Mesenchymal marker	Mouse anti-	0.1805556	
	CD73		
Sendai virus marker	Mouse anti-	0.7361111	
	Sendai virus		
Secondary antibody	Goat anti-	0.3888889	
	Mouse IgG2a		
Secondary antibody	(Alexa Fluor^®®^ 488)	0.3888889	
	Goat anti-Mouse IgG2b		
Secondary antibody	(Alexa Fluor^®®^ 488)	0.3888889	
	Goat anti-Mouse IgG3		
Secondary antibody	(Alexa Fluor^®®^ 488)	0.3888889	
	Goat anti-Rabbit IgG H+L (Alexa Fluor^®®^ 546)		
Secondary antibody	Goat anti-Mouse IgG1	0.3888889	
	(Alexa Fluor^®®^ 546)		

**Table 3:** Characterization of iPSC lines derived from the donors affected with DRVT/PS carrying *SCN1A* pathogenic variants.

iPSC Line	*SCN1A* Mutation (NM_001165963.4)	Disease	Gender	Ethnic Background
DS1	c.2589+2dupT	Dravet syndrome	Female	Caucasian
DS2	p.Arg1596His	FS/FS+/Panayiotopoulos syndrome	Female	Caucasian
DS3	p.Ser1516*	Dravet syndrome	Male	Caucasian

**Table 4:** Classification and validation of iPSC lines derived from the donors affected with DRVT/PS carrying *SCN1A* mutations.

Classification	Test	Results	Data
Mutation analysis	Sequencing	DS1: confirmation of splicing c.2589+2dupT/p.? mutation in the *SCN1A* gene;	Figure 1
		DS2: confirmation of missense c.4787G>A/p.Arg1596His mutation in the *SCN1A* gene;	
		DS3: confirmation of nonsense c.4547C>A/p.Ser156* mutation in the *SCN1A* gene	
Morphology	Microscope	All iPSC lines expressed the pluripotency markers: OCT4, SOX2, NANOG, and SSEA4; no presence of Sendai virus. Scale bars: 100 μm	Figure 2A
Phenotype	Immunocytochemistry	All iPSC lines have normal morphology. Scale bars: 100 μm	Figure 2B,C
	RT-PCR	All iPSC lines expressed high levels of pluripotency markers such as *OCT4*, *SOX2*, and *NANOG* comparable to hiPSC positive control, whereas HDFs of origin showed no expression	Figure 2D
Differentiation potential	Trilineage	All iPSC lines differentiated into the three germ layers including ectoderm (Otx2, SOX1), mesoderm (Brachyury, HAND1), and endoderm (GATA4, SOX-17). Scale bars: 100 μm	Figure 2E
	differentiation		
Genotype	Karyotype (G-banding)	DS1: 46, XX;	Figure 2F
		DS2: 46, XX;	
		DS3: 46, XY	
Microbiology/Virology	Mycoplasma testing	Negative	

## Data Availability

Not applicable.
